# BrainFD: Measuring the Intracranial Brain Volume With Fractal Dimension

**DOI:** 10.3389/fnagi.2021.765185

**Published:** 2021-11-26

**Authors:** Ghulam Md Ashraf, Stylianos Chatzichronis, Athanasios Alexiou, Nikolaos Kyriakopoulos, Badrah Saeed Ali Alghamdi, Haythum Osama Tayeb, Jamaan Salem Alghamdi, Waseem Khan, Manal Ben Jalal, Hazem Mahmoud Atta

**Affiliations:** ^1^Pre-Clinical Research Unit, King Fahd Medical Research Center, King Abdulaziz University, Jeddah, Saudi Arabia; ^2^Department of Medical Laboratory Technology, Faculty of Applied Medical Sciences, King Abdulaziz University, Jeddah, Saudi Arabia; ^3^Department of Informatics and Telecommunications, National and Kapodistrian University of Athens, Athens, Greece; ^4^Department of Science and Engineering, Novel Global Community Educational Foundation, Hebersham, NSW, Australia; ^5^AFNP Med Austria, Vienna, Austria; ^6^MRI Department, 251 General Airforce Hospital, Athens, Greece; ^7^Department of Physiology, Faculty of Medicine, King Abdulaziz University, Jeddah, Saudi Arabia; ^8^The Neuroscience Research Unit, Faculty of Medicine, King Abdulaziz University, Jeddah, Saudi Arabia; ^9^Division of Neurology, Department of Internal Medicine, King Abdulaziz University, Jeddah, Saudi Arabia; ^10^Department of Diagnostic Radiology, Faculty of Applied Medical Sciences, King Abdulaziz University, Jeddah, Saudi Arabia; ^11^Department of Radiology, King Abdulaziz University Hospital, King Abdulaziz University, Jeddah, Saudi Arabia; ^12^Department of Clinical Biochemistry, Faculty of Medicine, King Abdulaziz University, Rabigh, Saudi Arabia

**Keywords:** aging, biomarkers, fractal dimension, intracranial brain volume, MRI, neuroinformatics, OASIS brain database, VoxelMorph

## Abstract

A few methods and tools are available for the quantitative measurement of the brain volume targeting mainly brain volume loss. However, several factors, such as the clinical conditions, the time of the day, the type of MRI machine, the brain volume artifacts, the pseudoatrophy, and the variations among the protocols, produce extreme variations leading to misdiagnosis of brain atrophy. While brain white matter loss is a characteristic lesion during neurodegeneration, the main objective of this study was to create a computational tool for high precision measuring structural brain changes using the fractal dimension (FD) definition. The validation of the BrainFD software is based on T1-weighted MRI images from the Open Access Series of Imaging Studies (OASIS)-3 brain database, where each participant has multiple MRI scan sessions. The software is based on the Python and JAVA programming languages with the main functionality of the FD calculation using the box-counting algorithm, for different subjects on the same brain regions, with high accuracy and resolution, offering the ability to compare brain data regions from different subjects and on multiple sessions, creating different imaging profiles based on the Clinical Dementia Rating (CDR) scores of the participants. Two experiments were executed. The first was a cross-sectional study where the data were separated into two CDR classes. In the second experiment, a model on multiple heterogeneous data was trained, and the FD calculation for each participant of the OASIS-3 database through multiple sessions was evaluated. The results suggest that the FD variation efficiently describes the structural complexity of the brain and the related cognitive decline. Additionally, the FD efficiently discriminates the two classes achieving 100% accuracy. It is shown that this classification outperforms the currently existing methods in terms of accuracy and the size of the dataset. Therefore, the FD calculation for identifying intracranial brain volume loss could be applied as a potential low-cost personalized imaging biomarker. Furthermore, the possibilities measuring different brain areas and subregions could give robust evidence of the slightest variations to imaging data obtained from repetitive measurements to Physicians and Radiologists.

## Introduction

The quantitative measurement of the human brain volumes using segmentation software is highly correlated with the monitoring of neurodegeneration disorders (Jack et al., 2000; Kovacevic et al., 2009; Alexiou et al., 2017, 2019, 2020; Mantzavinos and Alexiou, 2017; Chatzichronis et al., 2019). Therefore, a few algorithms and online MRI databases are available for the assessment of the brain structure, measuring intrasessions and intersessions for the same subject, also applying procedures for potential manual repositioning differences from longitudinally acquired MRI, identification of artifacts, and segmentation errors (Dale et al., 1999; Fischl et al., 2002; Brewer et al., 2009; Fischl, 2012; Maclaren et al., 2014). While a reliable brain volume decline can be characterized as unbiased if and only if the loss is large enough (Narayanan et al., 2020), and by taking into consideration that brain lesions and brain atrophy associated with mild cognitive impairment (MCI) are higher than the expected decline per year in non-demented older adults (Liu J. Z. et al., 2003; Liu R. et al., 2003; Fotenos et al., 2005, 2008; Maclaren et al., 2014), any sources of high statistical variation, due to technical or physiological fluctuations, could be crucial for the reliability of the MRI results as a dementia biomarker (Caramanos et al., 2010; Fonov et al., 2010; Borghi and Van Gulick, 2018; Narayanan et al., 2020). In contrast, recent clinical studies suggest that the cortical functional connectivity networks show fractal properties and that any fluctuations to the fractal dimension (FD) of the brain gray and white matter are highly correlated with cognitive decline (Ha et al., 2005; Im et al., 2006; Li et al., 2007; King et al., 2009; Mustafa et al., 2012; Varley et al., 2020).

Biological structures similar to the brain gray matter usually have rough surfaces and are characterized by heterogenicity and self-similar structures. This mathematical self-similarity is the repetitive display of the whole structure after lowering the scaling. When the scale is altered, the structures are changed repeatedly, implying that the biological networks follow self-similarity patterns (Cerofolini et al., 2008). As the scale diminishes, the fractal becomes more complex (Kazemi Korayem et al., 2018). The definition of FD gives the complexity of these structures. The FD can reveal the properties and the mechanisms for the roughness of a structure. For example, if a fractal shape has a dimension of 2.3, it is more simplified than a 3D cube but more complicated than a 2D square. This non-Euclidean approach can be applied to minimize errors in the visualization of brain gray matter. One of the most common algorithms for calculating the FD and detecting image details is the box-counting algorithm. First, the fractal object is covered by a grid structured with small boxes of equal size. Each box should contain at least one region belonging to the fractal. Then, the number of boxes is counted. The next step is to design the grid again with the same properties but smaller square boxes. The abovementioned procedure is the traditional method where no lattices between squares or overlaps exist (Yadav and Nishikanta, 2010).

**FIGURE 1:**
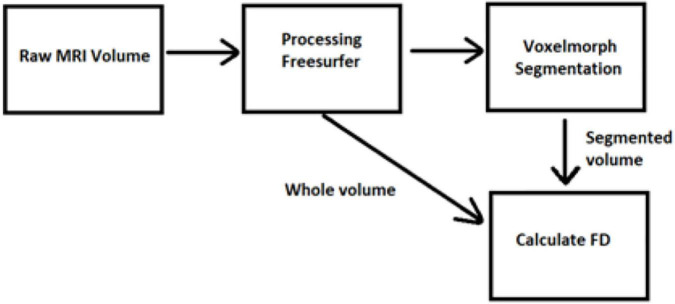
The algorithm of the proposed method – software.

**FIGURE 2:**
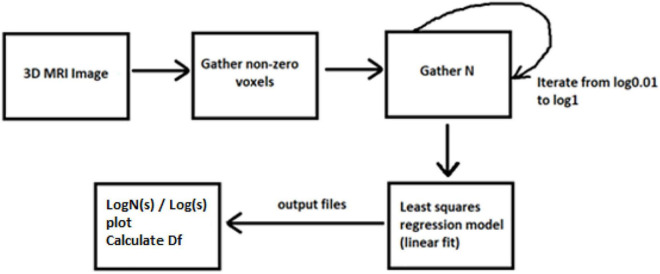
Visualization of computing fractal dimension (FD).

In amyotrophic lateral sclerosis (ALS), it is proved that the FD of brain white matter skeleton and general structure is significantly different between ALS patients with corticospinal tract hyperintensity and those with frontotemporal dementia groups (Rajagopalan et al., 2013; Di leva et al., 2015). Furthermore, while the numerical identification of differences in brain FD is still an open problem (Varley et al., 2020) and simultaneously could be a very strong evidence biomarker of consciousness disorder, we present new software in this study for handling MRI images and measuring FD of brain volume. We trained a learning-based registration tool using data obtained from the Open Access Series of Imaging Studies (OASIS) database (Marcus et al., 2007, 2010). The latest version of this database, i.e., the OASIS-3 (LaMontagne et al., 2019), includes a longitudinal neuroimaging, clinical, cognitive, and biomarker dataset for normal aging and Alzheimer’s disease (AD) from 2,168 MRI sessions and 1,608 PET sessions to more than 100 participants (LaMontagne et al., 2019). The participants in this database were classified according to their Clinical Dementia Rating (CDR). The brain MRI imaging sessions include T1-weighted (T1w), T2-weighted (T2w), fluid attenuated inversion recovery (FLAIR), arterial spin labeled (ASL), susceptibility weighted imaging (SWI), time of flight, resting-state blood oxygenation level dependent (BOLD), diffusion tensor imaging (DTI) sequences, PET imaging from three different tracers, C-Pittsburgh compound B (PIB), amyloid imaging tracer (AV45), and fluorodeoxyglucose (FDG) (LaMontagne et al., 2019). In the following sections, the algorithms for measuring the intracranial brain volume using the FD will be described based on the MRI brain data (Figures 1, 2).

## Materials and Methods

There is a rapidly increasing interest in box-counting algorithms in biology and medicine, such as applying fractal geometry for efficient recognition and capture of circulating cancer cells (Zhang et al., 2013; Maipas et al., 2018) and the correlation between the fractal property distribution and aging (Varley et al., 2020). Fractals have the property of self-similarity in various scales, and when the scale is altered, the structures are changed repeatedly. Furthermore, biological structures present complex fractal patterns similar to brain structures (Hofman, 1991; Varley et al., 2020). Therefore, depending on the image resolution and noise, FD may evaluate the condition of specific tissues such as the cerebral cortex (Blanton et al., 2001; Todoroff et al., 2014). In addition, the FD can influence the neurodegeneration analysis progression (Carstensen and Franchini, 1993; Jelinek and Fernandez, 1998; Pereira, 2010). While brain white matter loss is one of the characteristic lesions during neurodegeneration and other related disorders, measuring any structural brain change with high precision will act as a very effective and low-cost personalized imaging biomarker. Additionally, any geometric structural brain changes could be an excellent factor in measuring variations in the structural neural plasticity, while FD measures the roughness of surfaces, and software can be used to calculate the FD of an image (Table 1).

**TABLE 1:** Fractal dimension calculation software.

Software	Approach	Main properties
**BENOIT**	Measures the FD and hurst exponent of datasets using various methods to analyze self-similar patterns and self-affine traces, also applying a white noise filter	There are two main versions, for Windows and Matlab. The input data formats can be only: For 1D traces and size-frequency data, text format or MS-Excel format, for 2D patterns: BMP files and for 3D objects (available only in Matlab) BMP files, where the 3D object should be represented as a number of 2D slices.
**Fracdim** (Fractal Dimension Java Applet)	Calculates the box-counting dimension using a Monte Carlo algorithm.	This Java Applet imports only a set of points in a CSV file or an image, but the user is prompt to supply an image that has been thresholded to show where the fractal pattern is.
**FracLac** (Fractal Dimension and Lacunarity, part of ImageJ)	Describes morphology details represented in binary or grayscale digital images, using mass and box-counting FD and multi-fractal analysis data	Provide calculations and graphs. This plugin works on binary images and grayscale images or grayscale images converted to RGB. The images must be thresholded before analysis to ensure that only the pixels of interest are assessed.
**Fractal analysis system for Windows**	Calculates FD by the method of box-counting after preprocessing	This software calculates FD from rjb, png, pcx, jpg, and thinning images after preprocessing.
**Fractal Dimension Estimator**	Measures the FD of a 2D image using the box-counting method	This software measures the FD of a 2D image using the box-counting method after applying an RGB threshold to convert the image into binary data.
**Fractaldim-package**	Estimates an FD of the given data, using different methods regarding the type of dimensional time series	The package provides tools for estimating the FD of 1d or 2d data.
**Fractalyse** (Fractal Analysis Software)	Computes the FD of the black and white image, curve, and network	It is a software application for computing FD of 2D bitmap images, vector images, and networks.
**Gwyddion**	A modular program for scanning probe microscopy (SPM) data visualization and analysis	It is a modular program for several SPM data formats visualization and analysis.
**HarFA** (Harmonic and Fractal Image Analysis)	Performs harmonic and wavelet analysis of digitized images and calculations their fractal parameters	The software provides tools for estimating the FD and other statistical parameters of 2D images.
**Hausdorff** (Box-Counting) **Fractal Dimension**	Returns the Hausdorff FD of an object represented by its binary image	A MATLAB module returns the Hausdorff fractal dimension of an object represented by a binary image.
**UJA-3DFD**	Computes the 3D FD from brain MRI, calculating the 3D box-counting of the brain’s entire volume and 3D skeletonization	This software calculates the FD in 3D images. However, it offers only FD calculation for the whole brain volume and not each brain region separately.
		

Compared to other tools, we succeeded in implementing the image segmentation of brain regions in a 3D fashion.

The box-counting algorithm is the most commonly used technique for FD measurement (Schaefer et al., 1991; Fernández-Martínez and Sánchez-Granero, 2014; Joosten et al., 2016; Soltanifar, 2021), calculating the Minkowski–Bouligand dimension as follows:


(1) $$D_{\text{box}}\,\left(S\right)={\text{lim}}_{g\to 0}\,\left(\frac{\text{log}\,N\,\left(\text{ε}\right)}{\text{log}\,\left(\frac{1}{\text{ε}}\right)}\right),$$

D_*box*_(S) is the FD of the box, *S* is the fractal, *N*(ε) is the number of boxes, and ε is the scaling factor. The parameter ε is computed with the formula $\varepsilon =\frac{1}{s}$ where *s* is the length of each box. The majority of related studies mainly introduce the FD calculation from 2D MRI images, while others apply the multifractal analysis to examine different dynamics in the system. In our case, we have only calculated FD with a box-counting algorithm for the 3D brain structures. By calculating a whole 3D image instead of 2D scans, we ensure better accuracy in results.

**FIGURE 3:**
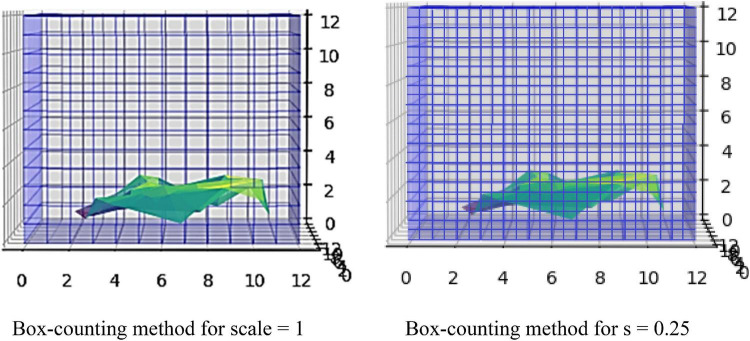
The algorithm calculating FD in 3D examples.

The concept of this algorithm in our study is to divide the 3D image into cubes. The 3D image includes a region of interest (ROI). Then, we calculate the number of *N* cubes with length ε of each box as part of the ROI. By applying equation (1) in each iteration, we calculate the FD. If the variable *D* in every iteration has a slight deviation, then logN/logs are stable, which means a linear correlation between logN and logs. In that case, the complexity of the structure can be efficiently described by FD. If not, the multifractal analysis is required to effectively describe the structure while other dynamics are included in the system in which FD cannot efficiently describe (Figure 3).

The data have been acquired from OASIS-3 (LaMontagne et al., 2019), which includes MRI (Berger, 2002; Grover et al., 2015; Chow et al., 2017) and PET (Willemsen and van den Hoff, 2002) data. We have used T1w MRI images (Chavhan et al., 2009; Yokoo et al., 2010), which have been later imported into the FreeSurfer (Fischl, 2012) for further image processing (Deserno Né Lehmann et al., 2013; Uchida, 2013). Then, the data were cropped to fit into the VoxelMorph software (Dalca et al., 2019) to perform image segmentation (Karsch et al., 2009; Despotović et al., 2015; Bui et al., 2017; Mondal et al., 2018; Buda et al., 2019; Dalca et al., 2019; Dolz et al., 2019; Rickmann et al., 2019, 2020; Cerri et al., 2020, 2021).

Our study has included 609 adults with normal cognitive ability (controls) and 489 multiple-staging dementia subjects aged from 42 to 95 years old, totaling 1,961 scan sessions. Each subject had multiple scan sessions, and each session included raw images from the MRI scanner. Some of them may have an additional file processed by FreeSurfer. Those files are already processed, so they are used in our next stage, avoiding image processing. The raw data obtained from the OASIS-3 brain dataset (LaMontagne et al., 2019) in the form of T1w 3D MRI images following the Neuroimaging Informatics Technology Initiative (NIfTI) format (Larobina and Murino, 2014), while the processed files by FreeSurfer follows the.mgz format. The FreeSurfer has been used for the initial image processing (Fischl, 2012), such as Motion Correction and Conform Non-Uniform intensity normalization, Talairach transform computation, Intensity Normalization, Skull Stripping, Linear volumetric registration, and CA Intensity normalization. The abovementioned methods export the data into the preprocessed images of (256, 256, 256) resolution. However, all the processing and post-processing steps of FreeSurfer were not executed to reduce time complexity, while VoxelMorph is more efficient for image segmentation. Furthermore, the image files are converted from.mgz to.npz format and then cropped to fit the (160, 192, 224) dimensions required to apply the VoxelMorph algorithm to the segmentation of brain volumes.

There are many segmentation algorithms based on supervised learning, but with high complexity, similar to the FreeSurfer. Their training procedures rely on labeled images, which means that they are accurate for specific scenarios. However, when a new dataset is tested with different contrast levels, additional training is required. Other methods include convolutional neural networks, which perform better during the testing, but they lose accuracy when changes occur in the intensity distribution of the tested images. In contrast, VoxelMorph is an UNet architecture using an unsupervised Bayesian approach, which tackles the drawbacks of those methods and has faster performance. To train a model, a brain atlas with multiple 3D MRI scans with no manual delineations is required. Instead, each voxel of the atlas has a vector with prior probabilities for each segmented label. Thus, when importing a new dataset with distinct unobserved contrast, the VoxelMorph algorithm does not require training the network again for new labels. Instead, it is automatically adapted due to its unsupervised nature. VoxelMorph has been mainly tested on T1w MRI scans. The brain structures delineated from VoxelMorph are the cerebral cortex, white matter, lateral ventricle, cerebellar cortex, white matter, thalamus, caudate, putamen, pallidum, brain stem, hippocampus, and amygdala. One exclusive session from each patient is used to train the model to avoid including dependent measurements.

## Results

**FIGURE 4:**
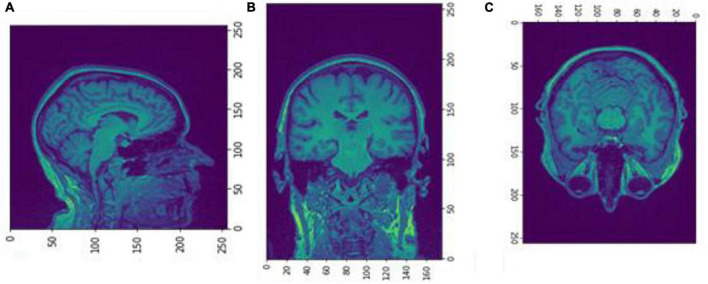
Slices **(A)** Sagittal, **(B)** coronal, and **(C)** axial planes. The output of FreeSurfer of session OAS30001_MR_d0129. This volume is imported in FreeSurfer and later exported as a.mgz file. The images are shown using NiBabel library version 3.2.1 (https://zenodo.org/record/4295521#.YCpJqeqxWMI).

**FIGURE 5:**
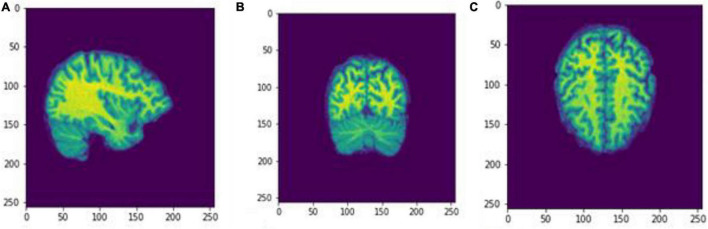
Slices **(A)** Sagittal, **(B)** coronal, and **(C)** axial planes. The output of FreeSurfer of session OAS30001_MR_d0129 as NIfTI files. This volume shall be imported later as a cropped image.npz file into the Box-Counting FD calculator. The images are shown using NiBabel library version 3.2.1 (https://zenodo.org/record/4295521#.YCpJqeqxWMI).

We executed two experiments. The first was a cross-sectional study that separated data into two CDR classes, 0 and greater than 1. VoxelMorph is used to train a model for each class. In the second experiment, we trained a model on multiple heterogeneous data and evaluated the FD development for each subject through multiple sessions. Random subjects have been chosen, and their development through FD metrics was assessed. In the following tables and figures for the OAS30001_MR_d0129 session, the whole process is implemented. The id of the session is defined from the id of the patient, i.e., 30001 and 0129, in the days since the subject was included in the study. OAS stands for OASIS database and MR for MRI. The raw data images from the MRI scan were imported on the software (Figure 4). The output is shown in Figure 5. We imported the images into VoxelMorph with a pretrained model run in Kaggle Kernel (i.e., 120 epochs, batch size of 1, and 19 sessions) to distinguish the brain areas. These Kaggle Kernel parameters were chosen only due to the restrictions of memory.

**FIGURE 6:**
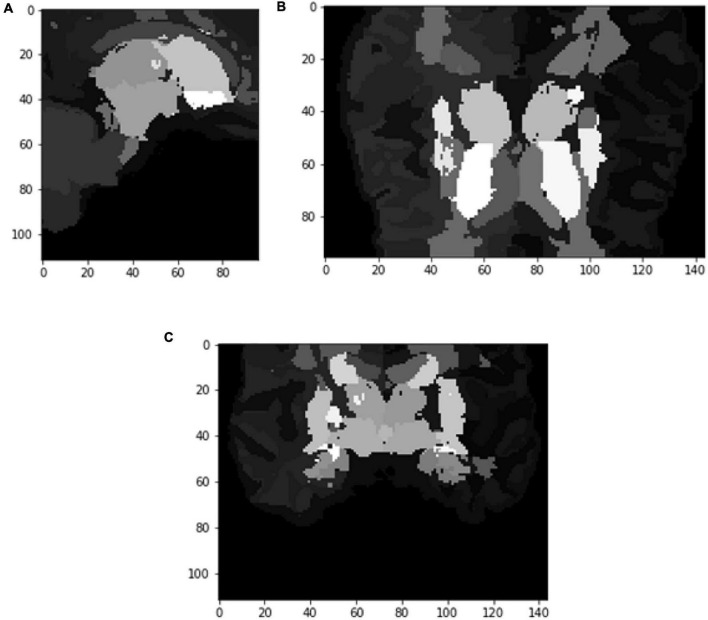
Slices **(A)** Sagittal, **(B)** coronal, and **(C)** axial planes. The output of VoxelMorph of session OAS30319_MR_d0043. Each brain structure has a unique label with grayscale values varying from 0 to 40. Later with filtering, each region is separated, and the Box-Counting FD is calculated. The images are shown using NiBabel library version 3.2.1 (https://zenodo.org/record/4295521#.YCpJqeqxWMI).

**FIGURE 7:**
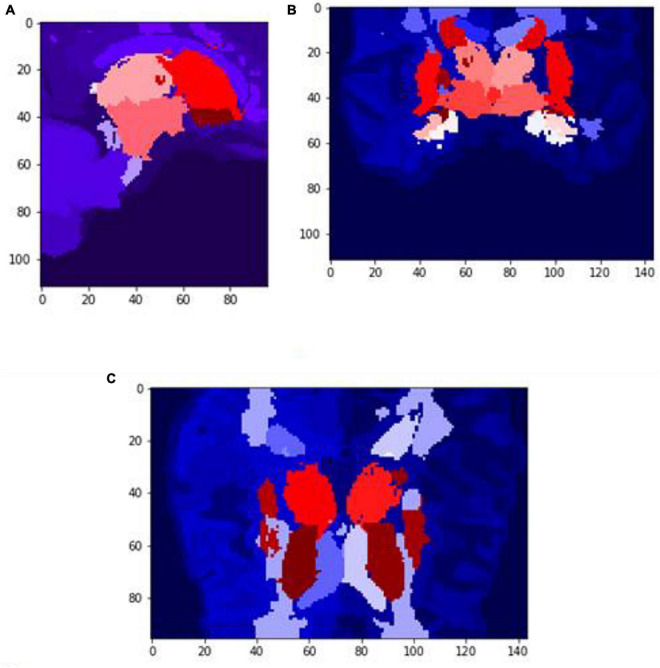
Slices **(A)** Sagittal, **(B)** coronal, and **(C)** axial planes. The output of VoxelMorph of session OAS30319_MR_d0043. Each brain structure has been colored differently.rgb values. The images are shown using NiBabel library version 3.2.1 (https://zenodo.org/record/4295521#.YCpJqeqxWMI).

**FIGURE 8:**
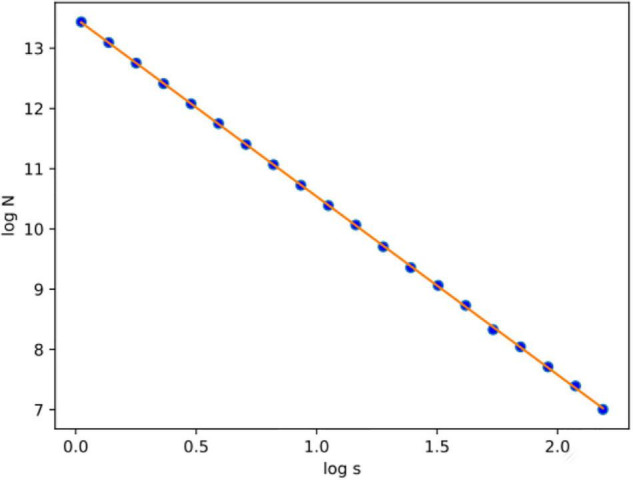
After calculating the logN/logs plot spots using the least square error, the fitted line is estimated for subject OAS30001_MR_d0129. Experiments have shown that at least 20 iterations were enough to estimate FD. Also, the linear relation of logN and logs suggests that FD efficiently describes the complexity of the structure; therefore, the multifractal analysis is not required. Root-mean-square error (RMSE) can describe how well a linear regression model describes the data. For data points close to the output line of the regression model, RMSE is expected to be low; therefore, the estimated FD is close to the theoretical solution. The two variables used in the linear regression model are the number of boxes (*N*) and the current scale (s). If a linear relationship describes the two variables, then the structure can be described by FD. If not, the structure is considered to have multifractal properties.

Figures 6, 7 show the segmented brain, with each region having a unique value ranging from 0 to 40. After segmentation, the FD is calculated for the brain regions, as shown in Table 2. Multiple experiments have shown that 20 iterations gave an efficient convergence to the box-counting FD, resulting in less time complexity (Figure 8). Iterations are related to the number of data points produced for each regression model. For example, 20 iterations correspond to 20 data points that are used to estimate the FD. During experimentation, it was observed that fewer iterations led to slightly different results. Using 20 or 25 iterations, the estimated FD was similar. However, more data points require more time to estimate. Therefore, choosing 20 data points is an efficient choice to balance time complexity and accuracy for the estimation of FD.

**TABLE 2:** The iteration steps to calculate the box-counting FD for the whole volume of session OAS30001_MR_d0129.

Scale	*N*
1.023292992280754	6.84523
1.146832521422478	4.85708
1.285286659943615	3.45522
1.440456010246376	2.45680
1.614358556826486	1.75729
1.809255910253820	1.26666
2.027682719521282	8.95650
2.272479635270844	6.40170
2.546830252585041	4.54770
2.854302513786581	3.25020
3.198895109691398	2.35480
3.585089482765549	1.63960
4.017908108489400	1.15920
4.502979812880891	8.62700
5.046612975635284	6.18900
5.655877570891540	4.14000
6.338697112569270	3.11100
7.103951700029557	2.23500
7.961593504173188	1.62600
8.922776195878269	1.10200

**FIGURE 9:**
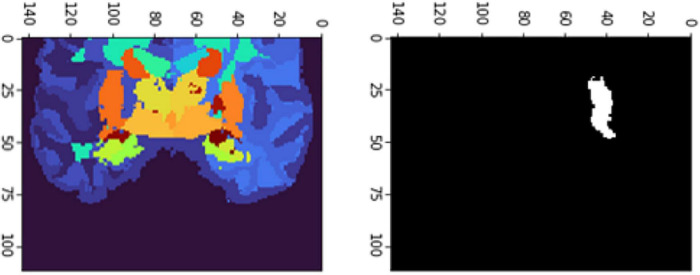
2D scan from the output volume of subject OAS30319_MR_d0043. We have chosen the third ventricle of the brain structure with an intensity value of 26.

**FIGURE 10:**
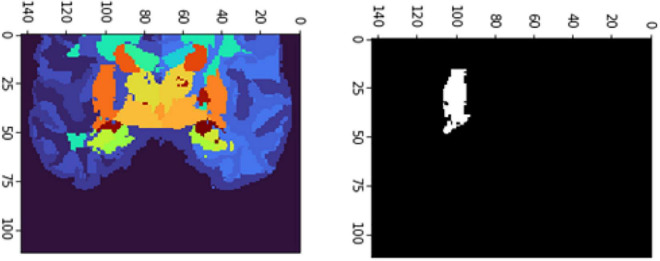
2D scan from the output volume of subject OAS30319_MR_d0043. We have chosen the right putamen of the brain structure whose intensity value equals 27.

The FD is calculated for the brain regions corresponding to 26 and 27 intensity values (Figures 9, 10) separately for four different subjects, namely, OAS30319, OAS30524, OAS31101, and OAS31170 (Table 3). In addition, the software has the functionality of measuring the FD for different subjects on the same brain regions, with high accuracy and resolution, offering the ability to compare brain data regions from different subjects and on multiple sessions.

**TABLE 3:** Box-counting FD testing on multiple sessions corresponding to various subjects.

Session	FD		
	
	Whole volume	Third ventricle	Right putamen
OAS30319_MR_d0043	2.95129	2.37498	2.59033
OAS30524_MR_d0198	2.95208	2.35327	2.50763
OAS31101_MR_d0076	2.95413	2.36024	2.52151
OAS31170_MR_d2410	2.95321	2.34761	2.54668

Extending the functionality described in Table 3, the software calculates and compares FD for the same subject and repetitive MRI measurements. This procedure is not yet presented widely (Krohn et al., 2019). Table 4 calculates the FD of the whole brain volume for the Third Ventricle and Right Putamen, respectively, of the same subject, OAS30109, and four different MRI sessions. OAS30109 is recorded at the initial clinical diagnosis as Female, Aged 72.27, Handedness Right, Education level 13, and Caucasian Race. During the repeated clinical evaluations for OAS30109 on days 1,102, 2,237, and 3,000, there was no report for MCI, dementia, or other neurological conditions, resulting in cognitive impairment or neuropsychological problems. We observed an average of 0.3% variation from the minimal variation of FD.

**TABLE 4:** Box-counting FD testing on multiple sessions of the same subject OAS30109.

Session	FD		
	
	Whole volume	Third ventricle	Right putamen
OAS30109_MR_d0270	2.946480299699088	2.3627751053697	2.5383689529296
OAS30109_MR_d0432	2.956639640069534	2.3414306913102	2.5583078971937
OAS30109_MR_d0997	2.957821984011597	2.3122620327539	2.5603106683312
OAS30109_MR_d2310	2.954899003322276	2.2987594704108	2.5215747308024

OAS30052 is also recorded at the initial clinical diagnosis as Female, Aged 59.02, Handedness Right, Education level 18, and Caucasian Race, diagnosed with depression. However, during the repeated clinical evaluations for OAS30052 on days 0728, 1,512, 2,650, and 3,028, there was no report for MCI, dementia, or other neurological conditions. Still, there was a report for depression, except for the clinical diagnosis on day 1,512.

**FIGURE 11:**
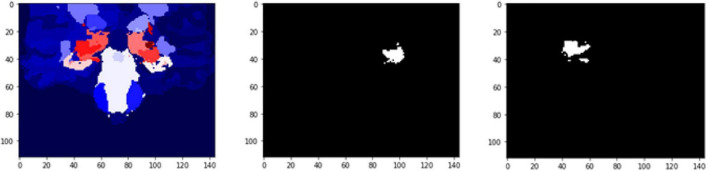
2D scan from the output volume of subject OAS30052_MR_d0693. Right Thalamus Proper and Left Ventral DC equal to 23 and 24 intensity values.

**FIGURE 12:**
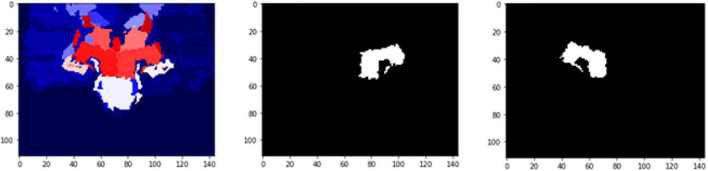
2D scan from the output volume of subject OAS30052_MR_d0693. We have chosen the Right Thalamus Proper and Left Ventral DC of the brain structures equal to 23 and 24 intensity values. The 2D scans are from different axial slices of the same subject compared with the 2D scans in Figure 11.

The results (Table 5) are verified from the corresponding variation of FD concerning the whole brain volume and the regions corresponding to Left Ventral DC and Right Thalamus Proper (Figures 11, 12). There was an increase of FD of more than 0.4% during the first and the second clinical assessment and again a continuous decrease. Figures 11, 12 correspond to 2D scans from different axial slices of the same subject, even though the FD calculation has been obtained, as in every case, from the 3D brain model for maximizing the accuracy. Thus, even though the pathophysiology of depression may be identified in many brain regions (Pandya et al., 2012), we have chosen the Right Thalamus Proper and Left Ventral DC as the subareas of the thalamus. Recent researches have revealed that brain areas implicated in depression are the amygdala, the hippocampus, and the thalamus, even though an exact formula of the correlation between volumetric abnormalities in these regions and the development of depression is not yet proved (Sheline et al., 1998; Kronmuller et al., 2008; MacQueen et al., 2008; Lorenzetti et al., 2009).

**TABLE 5:** Box-counting FD testing on multiple sessions of the same subject OAS30052.

Session	FD			
	
	Whole volume	Right thalamus proper	Left ventral DC	Clinical diagnosis
OAS30052_MR_d0693	2.95047009894	2.519209063	2.397232208	No MCI Depression
OAS30052_MR_d1296	2.9475385443	2.518800477	2.407069774	No MCI No Depression
OAS30052_MR_d2709	2.9478168557	2.505436960	2.383144328	No MCI Depression
OAS30052_MR_d2737	2.9484438042	2.500205780	2.384530171	No MCI Depression

This experiment removed repeated sessions from each subject to delete any within-subject factor and perform a cross-sectional study. As a cross-sectional study, each session corresponded to one patient and *vice versa*. The subjects were separated into the classes of CDR = 0 (control group) and CDR ≤ 1 (patients with at least mild dementia). MRI sessions were not labeled, while CDR sessions are usually performed on different dates from the MRI sessions. Therefore, to label each MRI session, some criteria had to be applied. First, each MRI session had to have occurred between two CDR sessions of the same score. If the MRI session took place between two different CDR sessions with different MRI scores, the score from the closest CDR session could be selected, considering that the chosen CDR session was performed no further than 45 days from the MRI session. In the MRI session being close to only one CDR session, the threshold of 45 days had again been chosen to ensure that the dementia level was correctly assessed. In any other scenario, subjects were dropped out of this study. The control group consisted of 593 subjects and 73 subjects with at least mild dementia.

After labeling the data, we trained a segmentation model using VoxelMorph for each class. Then, each volume was segmented into brain regions with its corresponding class model. Second, using Python and scikit-learn package (Pedregosa et al., 2011), we trained efficient support vector machine (SVM) classifiers in Left Ventral DC, Right Ventral DC, Right Putamen, and Brain Stem to diagnose subjects with at least mild dementia (CDR = 1) and to discriminate them from the control group. Other regions within the ROI did not show any significant differences. Finally, we used SVM models with radial basis function (RBF) kernels to find the margin between the two classes and evaluated the results with five-fold validation. Figures suggest that even measurement with one variable, either volume or FD, subjects can be accurately classified with a precision of 100%.

**FIGURE 13:**
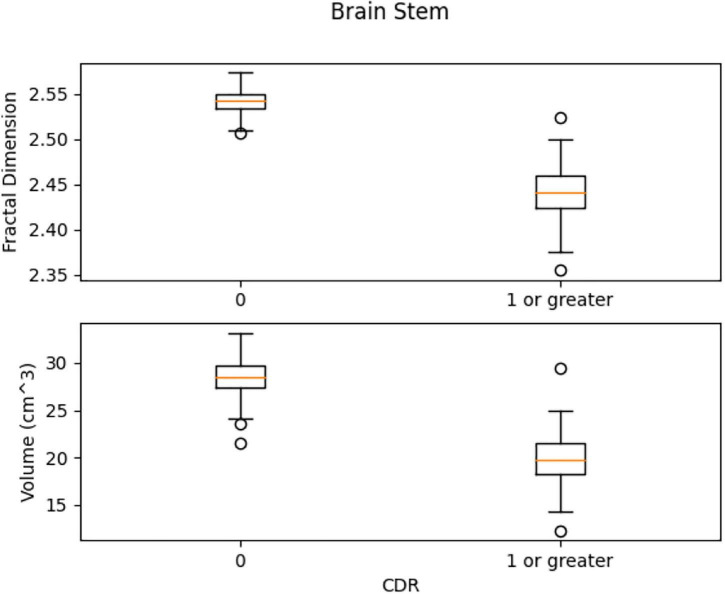
Comparison of the distribution of health vs. control group. There is a slight overlap of the two classes in both features.

**FIGURE 14:**
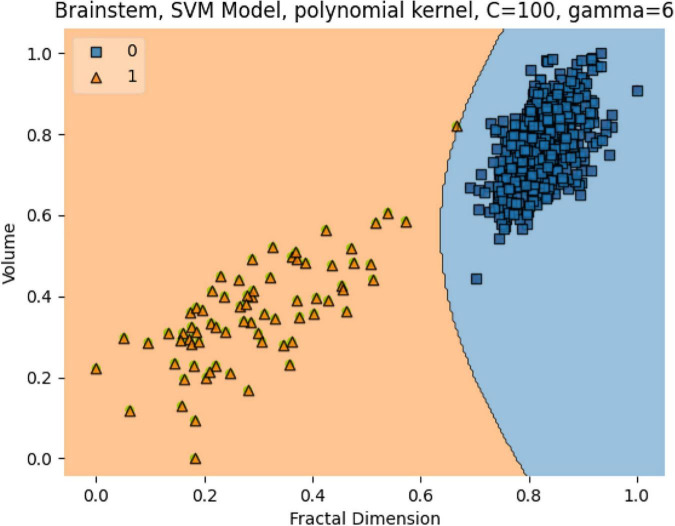
The SVM model separating the two groups. Features are Volume and FD. Hyperparameters are regularization hyperparameter C = 100 and gamma = 6 with polynomial kernel.

**FIGURE 15:**
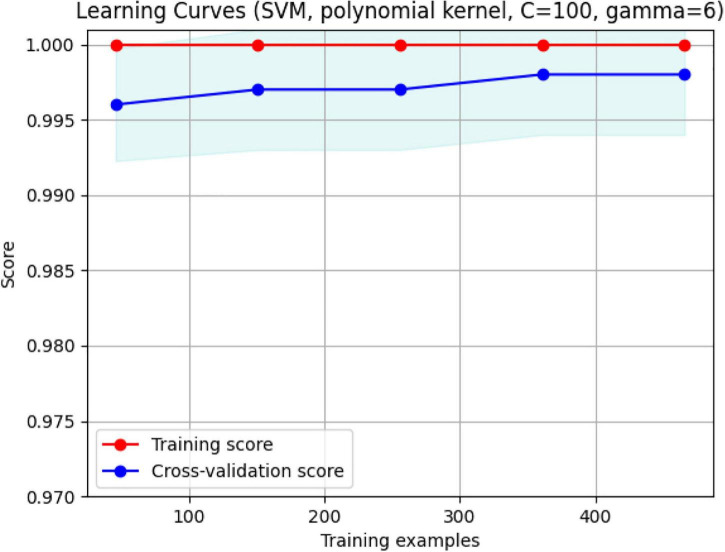
The validation plot. It shows that for a training sample of approximately 480, maximum accuracy with no overfitting is achieved. With FD and Volume, the two groups are separated efficiently with 99.5% accuracy.

In Figure 13, the box plots imply an overlap of the volumetric and fractal measurements of the brain stem. Two linear SVM models were trained. Volume and FD were used as features for each case. It is shown that the FD feature discriminates more efficiently between the two classes (Figures 14, 15). The increased nature of the decision boundary may imply a constant increase in FD during the years.

**FIGURE 16:**
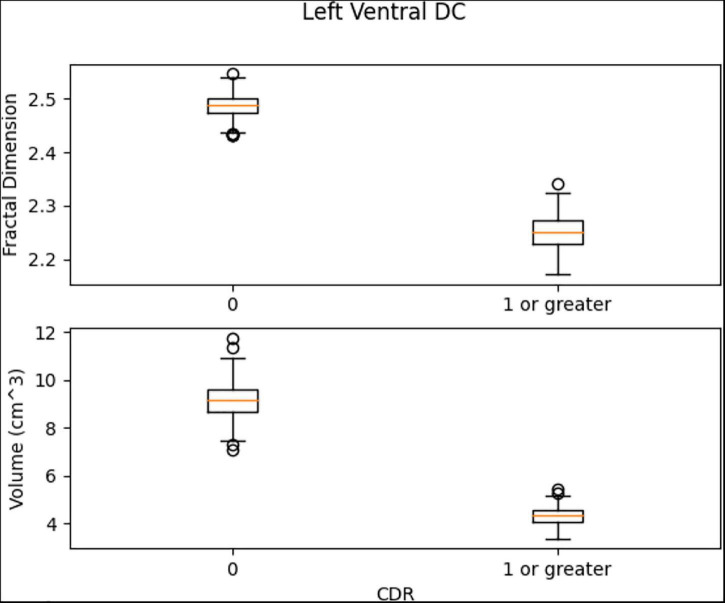
The box plots for Left Ventral DC region.

**FIGURE 17:**
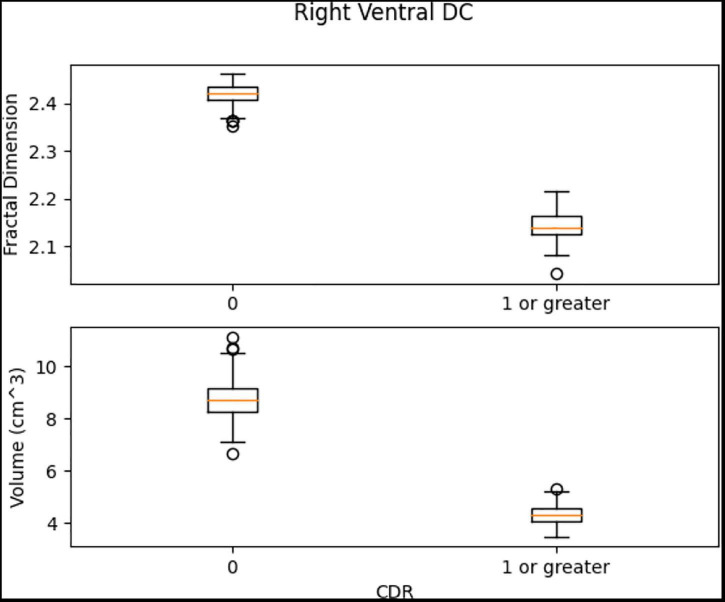
The box plots for Right Ventral DC region.

**FIGURE 18:**
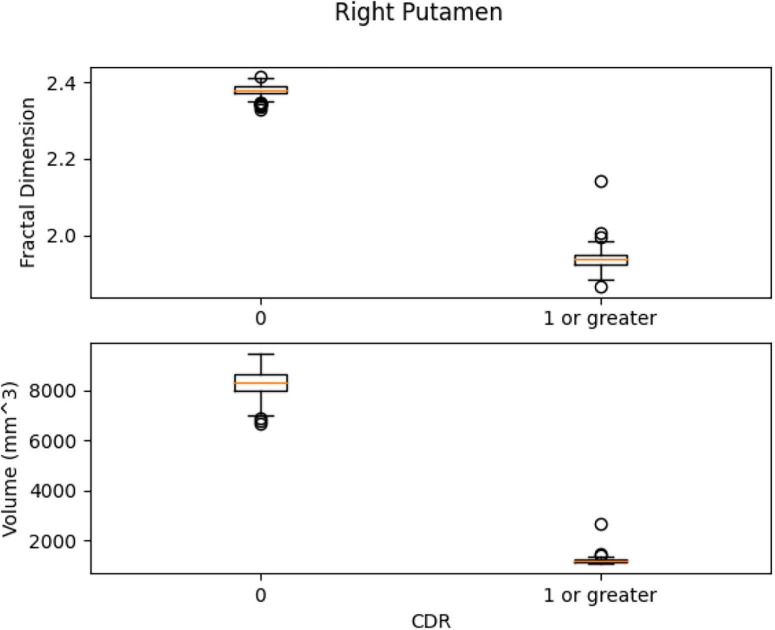
The box plots for Right Putamen.

Left Ventral DC, Right Ventral DC, and Right Putamen box plots have shown significant differences in volume and FD (Figures 16–18). Linear SVM classifiers using volume and FD as features achieve 100% precision and recall due to the vast distance between the two groups.

**FIGURE 19:**
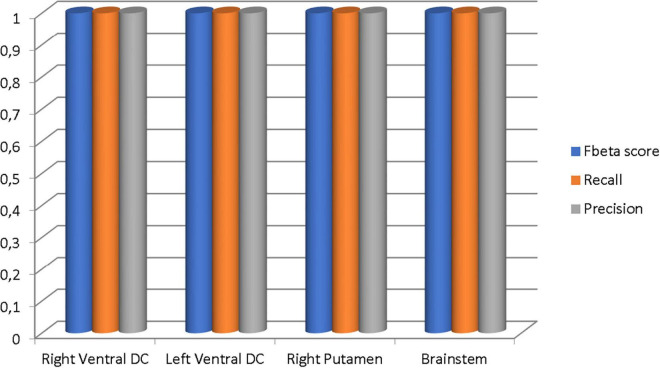
The Fbeta score, recall, and precision metrics of the classifications performed in each brain region.

**FIGURE 20:**
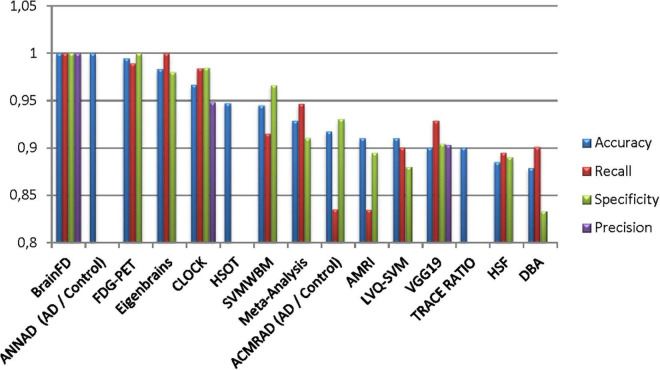
Comparison of the current classification (BrainFD) with other methods. Each compared method corresponds to the following bibliography, respectively (Huang et al., 2008; Kloppel et al., 2008; Magnin et al., 2009; Desikan et al., 2009; Lopez, 2009; Ortiz et al., 2013; Dukart et al., 2013; Yong et al., 2014; Bharanidharan and Rajaguru, 2018; Chen et al., 2020).

**FIGURE 21:**
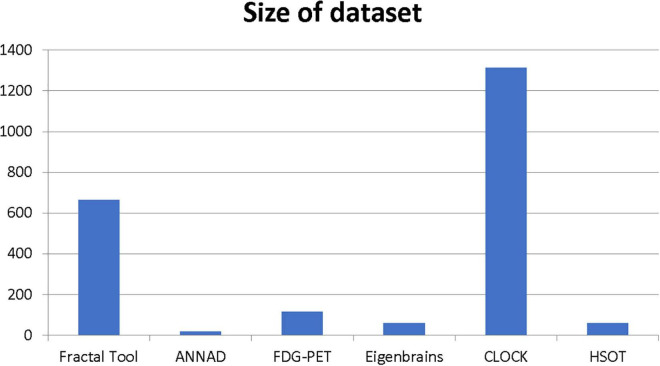
Size of the dataset of the most efficient methods.

Our results suggest state-of-the-art performance in classifying healthy and dementia subjects, as shown in Figure 19. Since the dataset was unbalanced and the control group had a size of 8.25 greater than the dementia class, the Fbeta score metric has been used with factor β = 8.25 instead of precision. Another reason for penalizing more the errors in the dementia class is that it is significant to minimize type I error by maximizing recall. Furthermore, it is crucial to identify the patient group efficiently in medical problems and avoid assigning a patient wrongly to the control group. In Figures 20, 21, a comparison of our classification with the other existing methods is displayed. Our method outperforms all other methods in terms of the accuracy and size of the dataset. More precisely, although the first three algorithms have similar performance with our classification, they yield results from smaller datasets.

## Conclusion

This study presents a new software using the OASIS brain database for brain volume measurement using the definition of FD. The possibilities measuring different brain areas and subregions could give robust evidence of the slightest variations to imaging data obtained from repetitive measurements to Physicians and Radiologists.

Three experiments on the OASIS brain dataset have shown the use of FD as a tool for the diagnosis and prediction of early dementia. The first experiment trained the segmentation model on a heterogeneous dataset to ensure its universal validity for repeated measurements. The second experiment showed different imaging profiles on the early stages of dementia. The third is a classification to distinguish healthy subjects from patients of various dementia stages. The latter shows high accuracy compared to any existing method.

Each segmentation model is trained on an online Kaggle Kernel with a 16 GB NVIDIA TESLA P100 GPU. The primary limitation of the procedure is that the algorithm required more memory to allocate images of (160, 192, 224) resolution; therefore, we manually reduced them. The corresponding atlas to (144, 112, 96) dimensions the specific regions of the brain. Due to these memory constraints, the model has also been executed with a batch size of 1 and 120 epochs for both experiments. The programming code for the FD calculation can be found in the following GitHub link: https://github.com/BrainLabVol/BrainFD and basic instructions of the Graphical User Interface in the Appendix.

Future study is planned to validate this model for control and dementia groups, using R correlation to validate the FD and more data to train and validate the cross-sectional study. Also, an improved computational system will perform the segmentation of the whole brain volume instead of an ROI and will adequately evaluate the trained segmentation model.

## Data Availability Statement

Publicly available datasets were analyzed in this study. This data can be found here: https://www.oasis-brains.org/. The programming code for the FD calculation can be found here: https://github.com/BrainLabVol/BrainFD.

## Ethics Statement

Ethical review and approval was not required for the study on human participants in accordance with the local legislation and institutional requirements. The patients/participants provided their written informed consent to participate in this study.

## Author Contributions

All authors made a significant contribution to the study reported, whether that is in the conception, study design, execution, acquisition of data, analysis, and interpretation, or in all these areas, took part in drafting, revising, or critically reviewing the article, and gave final approval of the version to be published.

## Conflict of Interest

The authors declare that the research was conducted in the absence of any commercial or financial relationships that could be construed as a potential conflict of interest.

## Publisher’s Note

All claims expressed in this article are solely those of the authors and do not necessarily represent those of their affiliated organizations, or those of the publisher, the editors and the reviewers. Any product that may be evaluated in this article, or claim that may be made by its manufacturer, is not guaranteed or endorsed by the publisher.

## Documentation

### Installation guide

Necessary libraries:

– pip install VoxelMorph
– pip install TensorFlow

**Table:** GUI

Option	Description
Single File	Manipulate a single file
Choose subject	Select a file
Raw Data	Select a NIfTI, DICOM file to perform Image Segmentation with FreeSurfer
Fs file (Norm.mgz)	Load a mgz file, this file is extracted after Image Segmentation
Fs file (npz)	Load a npz file that has been previously converted from mgz to npz
Vxm file	Load the output npz file that VoxelMorph outputs after segmentation
Fd file	Load the npz file produced after Fractal analysis
Mgz to npz	The mgz file is converted into npz format
VoxelMorph	After importing npz files, perform image segmentation with VoxelMorph
Estimate Df and L	Estimate Fractal Dimension of the imported npz file

**Table:**

Option	Description
Multiple Files	Manipulate multiple files
Choose subjects	Select a parent folder containing NIfTI or DICOM files
Raw Data	Select a folder with NIfTI, DICOM files to perform Image Segmentation with FreeSurfer
Fs file (Norm.mgz)	Load a folder with mgz files, those files are extracted after Image Segmentation
Fs file (npz)	Load a folder with npz files that have been previously converted from mgz to npz
Vxm file	Load a folder with the output npz files that VoxelMorph outputs after segmentation
Fd file	Load a folder with the npz files produced after Fractal analysis
Mgz to npz	After importing mgz files convert them into npz format
Train a segmentation model with VoxelMorph	After importing npz files, train a segmentation model with VoxelMorph
Segment with VoxelMorph	Import previous trained model and previously imported npz files to perform image segmentation with VoxelMorph
Estimate Df and L	Estimate Fractal Dimension of the imported npz files, the output is a folder for each file
Create a dataset	Select a folder containing the output of Estimate Df and L, to create a dataset from multiple images

• The folder and the directories shall follow the hierarchical file structure of XNAT.
