# Erratum: BrainFD: Measuring the Intracranial Brain Volume With Fractal Dimension

**DOI:** 10.3389/fnagi.2022.852590

**Published:** 2022-02-04

**Authors:** Frontiers Production Office

**Affiliations:** Frontiers Media SA, Lausanne, Switzerland

**Keywords:** aging, biomarkers, fractal dimension, intracranial brain volume, MRI, neuroinformatics, OASIS brain database, VoxelMorph

Due to a production error, the reference for “Soltanifar, 2021” was incorrectly written as “Soltanifar, M. (2021). A generalization of the hausdorff dimension theorem for fractals. *Mathematics* 9:1546. doi: 10.1103/PhysRevE.85.056314”. It should be “Soltanifar, M. (2021). A generalization of the hausdorff dimension theorem for deterministic fractals. *Mathematics* 9:1546. doi: 10.3390/math9131546”.

The publisher apologizes for this mistake. The original version of this article has been updated.

